# Prevalence and nature of workplace bullying and harassment and associations with mental health conditions in England: a cross-sectional probability sample survey

**DOI:** 10.1186/s12889-024-18614-7

**Published:** 2024-04-24

**Authors:** Annie Bunce, Ladan Hashemi, Charlotte Clark, Stephen Stansfeld, Carrie-Anne Myers, Sally McManus

**Affiliations:** 1 Violence and Society Centre, City, University of London, Rhind Building, Northampton Square, London, EC1V 0HB UK; 2 Population Health Research Institute, St George’s, University of London, Cranmer Terrace, London, UK; 3 Centre for Psychiatry and Mental Health, Barts & the London School of Medicine, Queen Mary University of London, London, UK; 4 Department of Sociology and Criminology, School of Policy and Global Affairs, City, University of London, London, EC1V 0HB UK; 5 National Centre for Social Research, 35 Northampton Square, London, EC1V 0AX UK

**Keywords:** Workplace, Bullying, Harassment, Mental health, Mental wellbeing, Common mental disorder, Probability survey, Inequalities

## Abstract

**Background:**

Evidence on workplace bullying and harassment (WBH) in the UK has not used probability-sample surveys with robust mental health assessments. This study aimed to profile the prevalence and nature of WBH in England, identify inequalities in exposure, and quantify adjusted associations with mental health.

**Methods:**

Data were from the 2014 Adult Psychiatric Morbidity Survey, a cross-sectional probability-sample survey of the household population in England. Criteria for inclusion in the secondary analysis were being aged 16–70 years and in paid work in the past month (*n* = 3838). Common mental disorders (CMDs) were assessed using the Clinical Interview Schedule-Revised and mental wellbeing using the Warwick-Edinburgh Mental Wellbeing Scale. Analyses were weighted. We examined associations between past-year WBH and current CMD using multivariable regression modelling, adjusting for sociodemographic factors. Interaction terms tested for gender differences in associations. The study received ethical approval (ETH21220–299).

**Results:**

One in ten employees (10.6%, *n* = 444/3838) reported past-year experience of WBH, with rates higher in women (12.2%, *n* = 284/2189), those of mixed, multiple, and other ethnicity (21.0%, *n* = 15/92), and people in debt (15.2%, *n* = 50/281) or living in cold homes (14.6%, *n* = 42/234). Most commonly identified perpetrators of WBH were line managers (53.6%, *n* = 244/444) or colleagues (42.8%, *n* = 194/444). Excessive criticism (49.3%, *n* = 212/444), verbal abuse (42.6%, *n* = 187/444), and humiliation (31.4%, *n* = 142/444) were the most common types. WBH was associated with all indicators of poor mental health, including CMD (adjusted odds ratio [aOR] 2.65, 95% CI 2.02–3.49), and 11 of 14 mental wellbeing indicators, including lower levels of confidence (aOR 0.57, 0.46–0.72) and closeness to others (aOR 0.57, 0.46–0.72). Patterns of association between WBH and mental health were similar in men and women.

**Conclusions:**

These findings reinforce a need for more cohesive UK legislation against WBH; guidance on recognition of bullying behaviours for employees, managers, and human resources, focusing on prevention and early intervention, and increased awareness of the impact of WBH on mental health among health service practitioners. Limitations include reliance on cross-sectional data collected before pandemic-related and other changes in workplace practices. Longitudinal data are needed to improve evidence on causality and the longevity of mental health impacts.

**Supplementary Information:**

The online version contains supplementary material available at 10.1186/s12889-024-18614-7.

## Background

Workplace bullying and harassment has been a growing area of interest worldwide during the last decades [1]. Despite increasing interest in the topic, for over a decade there has been no random-sample survey of the prevalence of workplace bullying and harassment (WBH) in England, nor of its associations with assessed mental disorder in victims. While the links between bullying and mental health are well documented among school and university students [2], the wealth of research carried out within educational settings has yet to be conducted in the workplace context. Many adults spend half their waking lives at work, making the workplace a major and specific location for exposure to forms of interpersonal aggression [3]. Indeed, WBH “has been recognised as an important social problem for over four decades” and is “costly to the organisation and to the individual” [4]. Whilst the need for attention to the phenomenon of workplace bullying is uncontested, responses to the problem have been hampered by difficulties and inconsistencies in defining and measuring it [1].

### Defining workplace bullying and harassment

The terms bullying and harassment are often used interchangeably and comprise similar behaviours [5]. WBH encompasses a wide spectrum of behaviours, from physical violence and shouting to unwelcome remarks, persistent unwarranted criticism, spreading malicious rumours, regularly picking on or undermining someone, overloading people with work and denying someone training or promotion opportunities [5, 6]. A commonly agreed definition of WBH is provided by Nielsen and Einarsen (2018: 73), who state it as “situations where an employee repeatedly and over a prolonged time period is exposed to harassing behaviour from one or more colleagues (including subordinates and leaders) and where the targeted person is unable to defend him−/herself against this systematic mistreatment”. Thus, a defining characteristic of WBH is that the negative acts in question are repeated regularly; rather than isolated episodes or events, bullying takes the form of systematic aggressive behaviour repeatedly directed towards the victim/s [7]. WBH is primarily of a psychological nature, including non-behaviour and social exclusion [8]. WBH has been described in a wide range of occupations, including in healthcare settings [9], academia [10], the banking sector [11], and semi-professional sport [12].

### Responding to workplace bullying and harassment

Exposure to WBH can be highly debilitating and the need for intervention is recognised [13]. Cowie et al. [14] highlighted that bullying had become identified as a serious issue in the workplace context and “in many countries, trade unions, professional organizations, and human resources (HR) departments have become more aware over the last decade that behaviours such as intimidation, public humiliation, offensive name-calling, social exclusion, and unwanted physical contact has the potential to undermine the integrity and confidence of employees and reduce efficiency”. Following this, over a decade ago, the UK government initiated national projects aiming to ‘place the issue of bullying at work on employers’ agendas’ (e.g. 15), although there has been no major initiative since. Moreover, unlike research and interventions concerning bullying in educational contexts, WBH rarely takes a victim-focused perspective. Research into WBH has focussed on characteristics and qualities such as “promoting the work environment”, for example, “poor leadership”, “job demands”, “role ambiguity”, and “organisational change” to explain bullying behaviour, rather than trying to understand, manage and tackle the “actual protagonists” [16]. This lack of focus and understanding has likely contributed to the persistence of the problem.

### Prevalence of workplace bullying and harassment

A lack of consistent definition, and variable quality and quantity of national- or occupational-level samples, makes determining WBH prevalence challenging [3]. One review found estimates ranged from 1 to 50%, depending on measurement strategy, occupation or sector, and country [17]. A meta-analysis incorporating samples from 24 different countries and one multinational sample found an average prevalence of WBH of 14.6% [7]. Reference periods varied from current to lifetime, with the majority of studies reporting 6–12 months prevalence. A recent systematic literature review across several countries concluded that, independent of the estimation method used and the country being studied, WBH is widespread [1]. Up-to-date, country-specific estimates based on national populations are essential to enable meaningful cross-cultural comparison, and to inform country-level policy and programming. However, the last nationally representative study of WBH in the UK was over a decade ago.

Most UK studies to date have reported WBH prevalence ranging between 10 and 20%, depending on the methodologies employed [18]. One survey of over 70 organisations found that 10.6% of employees reported having been bullied in the past six months [19], and a survey of trade union members found that 34.5% reported being bullied in the last six months [20]. Representative studies with random samples have generally yielded lower prevalence rates. The most recent large-scale nationally representative survey of WBH in the UK, the 2008 Fair Treatment at Work survey, found that 5% of respondents reported having experienced WBH in the past two years (up from 4% in the previous survey) [21], rising to 7% when experiences of working for a former employer within the same reference period were included [15].

### Impacts of workplace bullying and harassment

The potential scale and often-hidden nature of WBH has prompted studies to consider the potential harms associated with it. WBH can cause severe physical, social, psychological and psychosomatic problems for victims [13]. Qualitative research suggests the health consequences of WBH can be lifelong, and the longer the bullying continues the lesser the chance of recovery for victims (e.g. [22]). Quantitative studies have found associations between WBH and poor sleep, depression [23], psychological distress, anxiety [24], posttraumatic stress disorder (PTSD) [25], common mental disorders [26, 27], suicidal ideation [28] and suicidal behaviour [29]. These associations have been reported in many countries, including Scandinavian [30] and other European countries [24, 31], North America [32], Australia [33], Japan [34], and China [35]. Research also demonstrates that the impact of WBH extends beyond the primary victim, affecting witnesses and organisational cultures and productivity [36].

Existing findings therefore highlight the association between WBH and mental health. However, these studies have focused on general, brief screening tools such as the General Health Questionnaire (GHQ-12, [37]), or else been limited to cases identified in health or social administrative records, or are not generalisable due to methodological issues such as using non-random samples or focusing on subgroups (e.g., geographic regions, occupations or levels of seniority). The most recent UK survey of WBH [15] was limited by asking questions related to general psychological distress which participants attributed to the bullying, rather than to specific symptoms or assessed mental health conditions. Furthermore, only asking those who reported bullying precluded any comparison between those who had and had not experienced WBH. To the best of the authors’ knowledge there have been no nationally representative population-based studies of the prevalence of WBH in England and its associations with mental health outcomes measured using robust clinical assessment.

### Aims of the current study

The current study extends the WBH literature by considering associations with both poor mental health and positive mental wellbeing. We use data from the Adult Psychiatric Morbidity Survey (APMS), a large general population survey of the mental health of adults in England. The APMS makes clinically valid assessments of mental health, taking a uniquely holistic approach to positive and negative mental health, and differences according to whether an individual has been bullied in the workplace. The aims of the current study were to estimate the overall prevalence of WBH among people in paid work in England, compare the prevalence of WBH between groups, i.e. by characteristics protected in law (gender, age, ethnicity, sexual identity) and socioeconomic factors (e.g. income, deprivation level), and examine the nature of WBH in terms of the forms it took and who it was perpetrated by. Finally, associations between WBH and poor mental health and mental wellbeing were examined, after adjustment for potential confounders. We hypothesised that those reporting WBH would have worse mental health and lower mental wellbeing.

## Methods

### Participants and procedure

The APMS 2014 covered England’s household population aged 16 and above, using a stratified, multistage random probability sampling design drawing on the national Small User Postcode Address File. This involved multiple stages: sampling primary sampling units (PSUs); addresses within selected PSUs; and one individual from each selected address. Data collection took place from May 2014 to September 2015, with verbal informed consent. The final sample comprised 7,546 individuals interviewed face-to-face in their own homes by trained interviewers, a response rate of 57%. Interviews averaged 90 min in length, although some lasted as long as three hours.

Computer-assisted personal interviewing (CAPI) was supplemented with some sensitive information collected using computer-assisted self-completion interview (CASI), where the participant used the interviewer’s laptop. Participants were informed beforehand that the interviewer would be unable to see the results of the self-completed parts of the interview.

### Measures

#### Main outcomes: poor mental health and mental wellbeing

Common Mental Disorders (CMDs), comprising depression and anxiety disorders, were assessed using the detailed Clinical Interview Schedule– Revised (CIS-R). The CIS-R was administered by CAPI, it is an interviewer administered structured interview covering the presence of non-psychotic symptoms. It was used to generate indicators for the presence of 14 types of CMD symptoms in the past month and the presence of any CMD in the past week [38]. Each section of the CIS-R considers one type of CMD symptom and opens with an item on presence of the particular symptom in the past month. By using an endorsement to this item, an inclusive threshold was applied allowing for a range of subtle indicators of low mood and anxiety to be examined. The full CIS-R comprised over 130 items, and operationalising International Classification of Diseases (ICD-10) diagnostic criteria also enabled the presence of any CMD to be established, thus clinical treatment need was also identified.

Posttraumatic stress disorder (PTSD) was screened for using a separate tool administered as part of the CASI, the civilian version of the PTSD Checklist (PCL-c), a 17-item measure covering the Diagnostic and Statistical Manual of Mental Disorders (4th Edition; DSM-IV) criteria for PTSD [39]. A positive screen was defined according to the developer’s instructions as 50 or more on the derived symptom severity score, provided items from each of the three DSM-IV criteria for PTSD (re-experiencing; avoidance and numbing; hyperarousal) were endorsed. As above, a positive screen was used to indicate probable PTSD. However, a positive screen does not necessarily mean the disorder was present, but that there were sufficient indicators to warrant further investigation.

The Warwick Edinburgh Mental Wellbeing Scale (WEMWBS) was used to measure mental wellbeing during the face-to-face part of the interview (CAPI). The WEMWBS is a 14-item scale of positively phrased statements covering feeling and functioning aspects of mental wellbeing, validated for the general population [40]. Each statement has five response options: ‘none of the time’, ‘rarely’, ‘some of the time’, ‘often’, and ‘all of the time’. A binary variable was created for responses to each statement (‘often’ and ‘all of the time’ (yes); all other options (no)). Using original response options (scores 1–5), total scores were also calculated by summing the 14 individual statement scores. Higher scores indicate higher levels of mental wellbeing. See Supplementary Table 1 for exact wording of each statement.

### Main exposure: workplace bullying and harassment (WBH)

Those aged between 16 and 70 years were asked during the self-completion CASI if they had done any paid work in the past month, either as an employee or self-employed. Paid work was defined as any work for pay or profit, including casual work (e.g. baby-sitting, running a mail order club). Self-employed people were included if they worked for their own business, professional practice, or farm for the purpose of earning a profit. Those who indicated that they were in paid work were asked (also in the CASI) if they had personally experienced bullying or harassment at work in the past twelve months. Those who reported experience of WBH were then asked ‘who was the person or people responsible’ and ‘what form does or did the bullying take’. A showcard listing options was used for these two items, with participants also able to provide ‘other’ responses which the interviewer could type in verbatim. More than one response option could be coded. (See Supplementary Table 1).

### Covariates

A range of individual and area level demographic and socioeconomic factors were selected to profile the sample and to adjust for in the analyses. These questions were all part of the face-to-face CAPI. Standard demographic questions identified gender (men, women), age (banded for analysis: 16–34, 44–54, 55–70), de facto marital status (single; married or cohabiting; separated, divorced or widowed) and self-ascribed ethnic group (White British; White Other; Black/Black British; Asian/Asian British, and Mixed, Multiple or Other). Socioeconomic context was captured using a variety of different indicators: housing tenure (owner-occupier, renting from a social landlord, renting from a private landlord), whether can afford to keep the home warm in winter (yes/no), and debt (defined as being ‘seriously behind in paying within the time allowed’ for any of a list of 15 items or that they had had their utilities disconnected in the past year). Housing tenure was selected as an indicator of financial insecurity, being unable to keep one’s home warm was selected as indicative of chronic poverty, while being behind with debt repayments is an indicator of acute financial stress that can affect people both in and out of poverty. A measure of income was available but was not used due to high levels of non-response to this item from participants. While the study did not collect information on the deprivation of the area where people worked, the deprivation of the area where they lived was available and was used as an indication of re-employment opportunities. Area-level deprivation was measured using quintiles of the ranked English Index of Multiple Deprivation (IMD) scores (Noble et al., 2019). IMD provides relative levels of deprivation across all small areas (also known as Lower-layer Super Output Areas) across England. These were ordered and quintiled for analysis.

Two other factors were controlled for due to their potential to disadvantage people in the workplace: (1) informal caring responsibilities because such a commitment might, for example, require individuals to leave work on time, and (2) whether English was the participants’ first language because those who are not native English speakers may experience discrimination as a result. Caring responsibilities were established with a question on whether the participant looked after, gave help or support to family members, friends, neighbours or others because they have a long-term physical or mental ill-health or disability or problems related to age, excluding anything related to paid employment as a carer (yes/no). Whether English was the participant’s first language was asked and binary coded (yes/no). The questionnaire and further methodological details are available elsewhere [41, 42].

### Data analysis

Analyses accounted for the complex survey design. Weights were used to take account of selection probabilities and non-response, in order to render results representative of the household population. Population control totals were obtained from the UK Office for National Statistics population estimates for age by sex and region. True (unweighted) sample sizes are presented. The analytic sample comprised only those participants who were aged 16 to 70 years when interviewed and who reported having been in paid work in the month prior to interview (*n* = 3,838).

Analyses were conducted using Stata 17 [43]. The extent of missingness was minor at less than 1% for each variable except PTSD (3%), missing cases were therefore excluded from the analysis. Chi-square tests examined whether there were differences between subgroups (see Tables 2 and 3). Unadjusted and adjusted logistic regressions were run to estimate the odds of experiencing each poor mental health and positive mental wellbeing indicator by bullying experience. A range of covariates were controlled for due to possible association with the outcome or exposure variables (gender, age, ethnicity, marital status, housing tenure, whether can keep home warm in winter, any debt, and area-level deprivation). These covariates were selected from those available in the APMS dataset due to their known associations with both mental health and experiences of employment. Results are reported as unadjusted (ORs) and adjusted odds ratios (aORs) with 95% CIs. Independent sample t-test (or student’s t-test) was used to compare two independent means (for those with and without experience of WBH) at *p* < 0.05.

**Table 1:** Prevalence of past-year workplace bullying and harassment (WBH) and sociodemographic characteristics of the sample

		All in paid work^a^ (*n* = 3838)		No WBH in past year (*n* = 3394)		WBH experienced in past year (*n* = 444)		
		n	Weighted %	n	Weighted %	n	Weighted %	*p*-value^b^
Total		3838	100	3394	89·4	444	10·6	
Characteristics								
Gender	Men	1651	53·2	1490	54·1	160	46·1	0·006
	Women	2189	46·8	1904	45·9	284	53·9	
Age group	16–34	1089	37·5	979	38·1	108	32·2	0·06
	35–54	1922	45·5	1675	44·9	247	51·1	
	55–70	829	16·9	740	17·0	89	16·6	
Ethnicity	White (British and other white)	3433	87·6	3040	87·7	391	86·1	0·02
	Black/Black British	119	3·2	108	3·4	11	2·2	
	Asian/Asian British	183	6·4	159	6·5	24	6·3	
	Mixed, Multiple, Other	92	2·7	77	2·4	15	5·3	
English as first language	Yes	3475	88·5	3076	88·7	397	86·0	0·2
	No	365	11·5	318	11·2	47	14·0	
Marital/ cohabitation status	Married/cohabiting	2385	66·0	2119	65·9	265	67·1	0·9
	Single	923	26·0	804	26·1	118	25·2	
	Divorced/separated/widowed	532	8·0	471	8·0	61	7·6	
Caring responsibilities	Yes	774	18·9	672	18·5	102	22·1	0·08
	No	3066	81·1	2722	81·5	342	77·9	
Housing tenure	Owner occupied	2591	66·0	2307	66·3	284	63·8	0·6
	Social renter	408	10·9	351	10·8	57	11·6	
	Private or other	818	23·1	719	22·9	97	24·5	
Neighbourhood deprivation ^c^	Least deprived areas	1644	41·3	1470	41·8	173	36·9	0·07
	Moderately deprived area	805	20·2	713	20·3	92	19·0	
	Most deprived areas	1391	38·5	1211	37·8	179	44·0	
Serious debt in past year	Yes	281	6·8	231	6·4	50	9·8	0·01
	No	3536	93·2	3145	93·5	389	90·2	
Can afford to keep home warm	Yes	3573	94·1	3175	94·4	396	91·8	0·04
	No	234	5·9	192	5·6	42	8·1	

^a^ Adults aged 16 and over living in households in England who were in paid employment in the week prior to data collection, Adult Psychiatric Morbidity Survey 2014

^b^ p-value for the association between each characteristic and being bullied in the workplace in the past 12 months prior to data collection

^c^ Least deprived areas comprise the two least deprived quintiles and most deprived areas comprise the two most deprived quintiles, based on ranking of area-level English Index of Multiple Deprivation scores

**Table 2:** The person, or people, who carried out the workplace bullying or harassment

Person responsible for the WBH ^a^	Total (*n* = 444)		Women (*n* = 284)		Men (*n* = 160)		Chi-square *p*-value ^b^
	n	W%	n	W%	n	W%	
Line manager or another manager	244	53·6	154	52·9	90	54·5	0·6
A colleague	194	42·8	127	42·8	67	42·8	0·5
Client or a customer	57	13·5	37	13·8	20	13·1	0·9
Member of the public	21	5·3	10	2·8	11	8·3	0·1
A member of Human Resources	15	2·8	5	1·2	10	4·6	0·01
A student	10	2·0	9	3·5	1	0·3	0·08

^a^ Of those who reported experiencing bullying in the past 12 months· Participants could choose more than one response option

^b^ p-value indicates whether there was a difference between men and women in reporting of who was responsible for the WBH that they experienced

To examine whether the patterns of association between experiences of WBH and mental health outcomes differed between women and men, multivariable logistic regression models with interaction terms (between gender and experience of WBH) were tested (using both additive and multiplicative methods). Potential confounders (e.g., age, ethnicity, marital status, housing tenure, whether can keep home warm in winter, any debt, and area-level deprivation) were included in these analyses. Since no significant interaction effect was found, using either additive or multiplicative methods, results from the logistic regression models were not presented separately for men and women.

## Results

### Characteristics of the sample

Of the 7,546 survey participants, 3,838 were aged 16 to 70 and were in paid employment in the month prior to data collection. Of these, 46.8% were female, 45.5% were aged 35 to 54 years, 12.4% were from an ethnic minority background, 88.5% reported that English was their first language, 66% were married, and 18.9% had caring responsibilities for someone due to old age or disability. Regarding the economic characteristics of the sample, 34.0% rented their home, 6.8% reported being seriously behind with repayments or had had their utilities disconnected in the past year (in debt), and 5.9% were unable to afford to keep their home warm in winter (Table 1).

**Table 3:** The form the workplace bullying or harassment took

Form of bullying or harassment ^a^	Total (*n* = 444)		Women (*n* = 284)		Men (*n* = 160)		Chi- square *p*-value ^b^
	n	W%	n	W%	n	W%	
Excessive criticism	212	49·3	125	43·7	87	55·9	0·04
Shouting or verbal abuse	187	42·6	118	40·0	69	45·0	0·8
Humiliation	142	31·4	89	31·9	53	30·9	0·7
Setting unrealistic targets	134	31·2	76	27·7	58	35·3	0·04
Constantly changing instructions	137	29·8	83	29·5	54	30·1	0·3
Threatening behaviour	92	24·0	46	15·8	46	33·5	0·02
Excessive workloads	110	24·0	65	21·7	45	26·7	0·2
Refusing reasonable requests (e.g. for leave or training)	98	21·9	52	16·0	46	28·8	0·01
Physical abuse	13	3·7	1	0·4	12	7·5	0·001
Sexual harassment	12	2·5	8	2·9	4	2·0	0·8
Cyberbullying	9	1·8	4	1·2	5	2·6	0·2

^a^ Of those who reported experiencing bullying in the past 12 months. Participants could choose more than one response option

^b^ p-value indicates whether there was a difference between men and women in the form that the reported WBH took

### Prevalence and inequalities in workplace bullying and harassment in England

One person in ten (10.6%, *n* = 444) in paid work reported having experienced bullying or harassment at work in the past year (Table 1).

Those who reported experience of WBH in the past year were more likely to be women, to identify with a ‘mixed, multiple, or other’ ethnicity, and to be in debt than those who had not reported bullying (Table 1). Those with experience of WBH were also less likely to be able to keep their home warm during winter compared with those who did not report WBH. Regarding other demographic and socioeconomic characteristics (age, marital status, caring responsibilities, housing tenure, neighbourhood deprivation, and first spoken language), no significant differences were found between those who reported WBH and those who did not.

### The nature of workplace bullying and harassment experienced

Participants who reported bullying most commonly identified the perpetrator as a line manager (53.6%), colleague (42.8%), or a client or customer (13.5%) (Table 2). Similar patterns were found for men and women.

‘Excessive criticism’ and ‘shouting or verbal abuse’ were the most common types of workplace bullying, reported by 49.3% and 42.6% of people with experience of WBH, respectively (Table 3). ‘Humiliation’, ‘setting unrealistic targets’, and ‘constantly changing instructions’ were each reported by almost a third of those with experience of WBH. ‘Threatening behaviour’ and ‘excessive workloads’ were reported by a quarter, and ‘refusing reasonable requests’ (e.g., for leave or training) was reported by a fifth (21.9%) of those who had experienced WBH. Physical abuse (3.7%) and sexual harassment (2.5%) were reported by a small minority of victims.

Compared to women, men with experience of WBH were more likely to report that this had taken the form of excessive criticism, unrealistic targets, threatening behaviour, refusing reasonable requests, and physical abuse.

### Workplace bullying and harassment and poor mental health

Those who reported experience of WBH within the preceding year were more likely to experience all indicators of current poor mental health (Table 4).

**Table 4:** Association between being bullied at work and indicators of poor mental health

	Whole sample (*n* = 3839) n(W%)	No bullying reported (*n* = 3394) n(W%)	Bullying reported (*n* = 444) n(W%)	OR (95%CI)	AOR (95%CI)^a^
CMD in past week	597 (14·7)	460 (12·9)	137 (29·4)	2·81 (2·16 − 3·66)	2·65 (2·02–3·49)
PTSD in past week	213 (6·3)	162 (5·7)	51 (11·1)	2·06 (1·42 − 2·99)	2·11 (1·42 − 3·12)
CMD symptoms present in past month:					
Fatigue	1474 (37·6)	1242 (35·9)	231 (51·9)	1·92 (1·52 − 2·43)	1·85 (1·46 − 2·34)
Concentration problem	808 (20·2)	653 (18·7)	154 (32·3)	2·07 (1·62 − 2·65)	1·96 (1·52 − 2·52)
Sleep problem	1470 (35·5)	1242 (34·2)	228 (46·6)	1·68 (1·34 − 2·11)	1·56 (1·25 − 1·96)
Irritability	1833 (47·4)	1551 (45·3)	282 (64·7)	2·21 (1·74 − 2·80)	2·19 (1·71 − 2·79)
Worried about physical health	1038 (26·2)	870 (24·9)	166 (36·5)	1·73 (1·37 − 2·19)	1·65 (1·30 − 2·10)
Feeling depressed	1274 (31·2)	1068 (29·7)	206 (44·1)	1·87 (1·49 − 2·34)	1·74 (1·39 − 2·20)
Worried about things more than needed	1254 (31·9)	1045 (30·1)	209 (47·4)	2·10 (1·68 − 2·62)	1·98 (1·57 − 2·50)
Felt anxious	1212 (30·3)	989 (28·0)	223 (49·4)	2·50 (2·00–3·13)	2·41 (1·91 − 3·04)
Compulsion	275 (7·2)	226 (6·7)	49 (12·0)	1·90 (1·30 − 2·80)	1·88 (1·25 − 2·81)
Obsessive thoughts	483 (12·6)	395 (11·8)	88 (19·4)	1·79 (1·35 − 2·39)	1·75 (1·30 − 2·35)

^a^ Odds ratios adjusted for gender, age, ethnicity, marital status, housing tenure, area-level deprivation, whether can keep home warm in winter, and serious debt

^b^ CMD (Common Mental Disorders): any of six depressive and anxiety disorders: generalised anxiety disorder, phobic disorder, panic disorder, obsessive and compulsive disorder, depression, and CMD not otherwise specified. Assessed using the Clinical Interview Schedule– Revised (CIS-R).

^c^ Using the PTSD-Checklist

The prevalence of any CMD was twice as high in those with past-year experience of WBH (29.4%) compared with those who did not report WBH (12.9%). Using multivariable logistic regression analysis (Table 4) to account for a wide range of socio-demographic characteristics, WBH remained strongly related to all poor mental health indicators. The strongest effect was for any diagnosed CMD (aOR: 2.65, 95%CI: 2.02–3.49). Prevalence of every individual CMD symptom indicator was elevated in those exposed to WBH, including concentration problems (32.3% cf. 18.7%), feelings of depression (44.1% cf. 29.7%), anxiety (49.4% cf. 28.0%), and obsessive thoughts (19.4% cf. 11.8%). After adjustment for the covariates, the observed aOR was particularly pronounced for anxiety (aOR: 2.41, 95%CI: 1.91–3.04). Those exposed to WBH were also twice as likely as others in paid work to screen positive for PTSD (11.1% cf. 5.7%).

### Workplace bullying and harassment and mental wellbeing

Those who reported experience of bullying (M = 50.9, SD = 8.6) had lower total mental wellbeing scores than those who did not report experience of bullying (M = 53.5, SD = 8.1), (t (3810) = 6.37, *p* < 0.0001) (Table 5).

**Table 5:** Association between workplace bullying and mental wellbeing indicators using the Warwick Edinburgh Mental Wellbeing Scale

Mental wellbeing indicators	Whole sample (*n* = 3839) n(W%)	No bullying reported (*n* = 3394) n(W%)	Bullying reported (*n* = 444) n(W%)	OR (95%CI)	AOR (95%CI)^a^
Feeling optimistic	2141 (56·9)	1910 (57·0)	230 (56·1)	0·96 (0·77 − 1·21)	0·98 (0·78 − 1·23)
Feeling useful	2673 (69·4)	2392 (70·1)	279 (63·4)	0·74 (0·58 − 0·94)	0·74 (0·58 − 0·94)
Feeling relaxed	1527 (41·3)	1392 (42·5)	133 (30·1)	0·58 (0·45 − 0·75)	0·62 (0·48 − 0·80)
Feeling interested in other people	2589 (66·1)	2318 (66·8)	269 (59·5)	0·73 (0·59 − 0·90)	0·72 (0·57 − 0·90)
Had energy to spare	1318 (37·4)	1184 (37·9)	132 (32·8)	0·80 (0·63 − 1·02)	0·86 (0·67 − 1·10)
Dealing with problems well	2737 (72·2)	2461 (73·3)	274 (62·4)	0·60 (0·48 − 0·75)	0·64 (0·51 − 0·80)
Thinking clearly	3005 (78·7)	2701 (79·7)	302 (69·7)	0·59 (0·46 − 0·74)	0·63 (0·50 − 0·80)
Feeling good about themselves	2371 (63·9)	2145 (65·0)	224 (54·1)	0·63 (0·51 − 0·78)	0·65 (0·53 − 0·81)
Feeling close to other people	2861 (76·0)	2577 (77·1)	282 (65·9)	0·57 (0·45 − 0·73)	0·59 (0·46 − 0·76)
Feeling confident	2509 (67·0)	2277 (68·5)	230 (54·1)	0·54 (0·44 − 0·67)	0·57 (0·46 − 0·72)
Able to make up own mind about things	3303 (86·1)	2942 (86·7)	360 (81·5)	0·67 (0·50 − 0·89)	0·70 (0·52 − 0·95)
Feeling loved	3147 (85·1)	2812 (85·7)	333 (79·8)	0·66 (0·50 − 0·86)	0·69 (0·53 − 0·89)
Interested in new things	2383 (62·9)	2108 (62·5)	273 (65·4)	1·13 (0·91 − 1·40)	1·13 (0·91 − 1·40)
Feeling cheerful	2642 (70·1)	2384 (71·4)	256 (58·8)	0·57 (0·46 − 0·71)	0·59 (0·47 − 0·74)
Mean (SD)	53·2 (8·2)	53·5 (8·1)	50·9 (8·6)	T-test (df) t-test (3810) = 6·37, *p* < 0·0001	

^a^ Odds ratios adjusted for gender, age, ethnicity, marital status, housing tenure, area-level deprivation, whether can keep home warm in winter, serious debt

At the individual level after adjusting for a wide range of sociodemographic characteristics, a significant association was found between most indicators of mental wellbeing and experience of bullying. Those who reported recent experience of bullying were at decreased odds of experiencing mental wellbeing, with aORs ranging from 0.57 (95% CI: 0.46–0.72) for feeling confident, to 0.74 (95% CI: 0.58–0.94) for feeling useful. Exceptions were feeling optimistic, having energy to spare, and being interested in new things, which were not significantly associated with experience of bullying.

## Discussion

### Workplace bullying and harassment matters to mental health and wellbeing

This is the first probability sample survey in England to examine WBH as a risk factor for mental disorder robustly assessed to diagnostic criteria. The results show that WBH is not only associated with general distress, but also with depressive and anxiety disorders severe enough to warrant health service intervention and treatment. Human resource (HR) personnel and others making decisions on whether to intervene in potential WBH situations must recognise this potential severe level of harm.

That WBH was associated with every indicator of poor mental health confirms extant international evidence [44]. The associations are pervasive - people are more likely to feel low as well as worried and anxious, and behavioural implications include struggling with sleep and social relationships. Our findings confirm strong links with anxiety, depression, psychological distress, and PTSD [25] - with prospective studies also finding positive associations of similar magnitude (e.g. 27) - and highlight the importance of comparatively understudied ‘lower-level’ indicators of poor mental health (e.g. concentration problems, obsessive thoughts).

As well as being more likely to feel bad, people who reported WBH were also less likely to report positive feelings and functioning, including feeling cheerful, thinking clearly, and being able to make decisions. This is in line with Bowling and Beehr’s [45] finding that WBH was negatively associated with positive emotions at work and self-esteem and Cassidy et al.’s [46] finding that WBH was negatively associated with optimism and self-efficacy. Other studies have shown associations between psychosocial workplace factors and poorer wellbeing suggesting that wellbeing is eroded as well as the risk of adverse mental health outcomes increased by such exposure [47, 48].

### Bullying and harassment is widespread in England’s workplaces

Our results reveal the high prevalence of WBH in the working population of England, with one in ten reporting experience of WBH in the past year. While studies are not directly comparable due to methodological differences, this is consistent with an increase in the prevalence of WBH in England, with the last representative study reporting a 5% prevalence rate over the past two years [15]. This is particularly important given that bullying is about a pattern of behaviour, not one-off or isolated instances of conflict, meaning the experience and its impacts are often long-lasting [1].

### There are pronounced inequalities in workplace bullying and harassment

Our results also confirm that women are more likely to report experience of WBH than men [32, 49]. Cultural factors, social power and gendered expectations in the workplace may partially explain gender differences [49]. Type of work, for example whether working in a female- or male-dominated occupation, is also likely to be important [50]. A higher proportion of WBH was reported by those who identified with a ‘mixed, multiple, or other’ ethnicity than other ethnic groups. This is in line with existing evidence indicating that ethnic minority employees are more likely to experience WBH and discrimination than majority groups, although previous studies have found Asian and Black employees to also be at higher risk [51, 52].

Those who reported bullying were more likely to be in a financially disadvantaged position. This suggests financial strain may be a risk factor for WBH, possibly due to the fact that those who are financially dependent on work may also have less options, less power to escape, and hold more junior positions within the workplace (see also [53]). Such power dynamics are also reflected in the finding that managers were the most commonly identified persons responsible for carrying out WBH, followed by colleagues. In other UK-based studies, managers were identified as responsible in 70–80% of bullying incidents, and colleagues in approximately in one-third [17, 27]. Taken together these findings suggest WBH may be driven and exacerbated by issues of inequality, power and hierarchical organisational structures, with line managers also potentially subject to undue workplace pressures and demands from above [54]. Reports of WBH may also coincide with performance concerns from managers [55]. Whilst behaviours intended as legitimate performance management activities can be misinterpreted as bullying by the employee, it is also possible that HR practitioners attribute managerial bullying behaviours to legitimate performance management practice to exonerate mangers and protect the organisation [56]. The complexity of workplace environments creates challenges for identifying, understanding and addressing bullying.

### Workplace bullying and harassment mostly comprises criticism and verbal abuse

Excessive criticism was the most commonly reported form of bullying or harassment, followed by shouting or verbal abuse, humiliation, setting unrealistic targets and constantly changing instructions. Unmanageable workloads and being shouted at have also been identified as the most prevalent bullying behaviours in the workplace in other UK based studies [31]. The types of bullying behaviours reported differed for men and women, consistent with previous findings that women may experience more non-physical forms of WBH such as rudeness, social exclusion and humiliation, whilst men are more likely to experience threatening and/or physical aggression [57, 58]. The gender of the perpetrator is likely to be important here and would be key to analyse in a future study.

### Limitations

As the first probability sample survey in England to examine WBH as a risk factor for robustly assessed mental disorder, its generalisability to the wider population is a strength. However, the prevalence of WBH may have been influenced by the way the WBH questions were worded/sequenced in the APMS. Fevre et al. [15] noted that short, direct bullying questions, such as the stem question used on the APMS, may risk some types of negative workplace behaviours of interest not being reported. Underreporting may also have occurred for various reasons, including stigma [59], lack of recognition/agreement that a behaviour was bullying [60], patterns of non-response, and the exclusion of those not in paid work in the past month [61]. Risk of selection bias is also a limitation of the current study. Only participants who were in paid employment in the past month were asked to report any experience of WBH in the past 12 months. It is possible that those experiencing more severe forms of bullying earlier may have dropped out of work prior to this past-month period and not returned to any employment (e.g. see [62]), and their experiences were not captured.

The cross-sectional nature of the study means a temporal relationship between exposure to WBH and the onset of mental health outcomes cannot be inferred. Longitudinal and prospective studies testing the reverse causation hypothesis have found that people with poor mental health can be at increased risk of subsequent WBH [44]. Thus, poor mental health may have predisposed participants to report WBH in the current study, consistent with negative affectivity related to poor mental health. However, the timeframe of both exposure (past year) and outcome variables (past 4 weeks) mitigates this concern to some extent. Collecting data from victims only also means it is difficult to capture the complexity of working environments and competing pressures on both employers and employees.

The current study is based on data collected in 2014. Data from a further survey in the APMS series will be available in 2025 and able to provide an updated picture of WBH in England which reflects the situation post the COVID-19 pandemic, in particular the increase in remote working. Finally, exploration of the effect of gender was limited because the 2014 APMS only asked about binary gender and did not ask the gender of the perpetrator. Furthermore, the sample, because it is a probability sample representative of the population in England which did not oversample from minority groups, was underpowered to explore ethnicity robustly. Small sample sizes in subgroups also limited the scope for disaggregation by ethnicity, sexual identity, occupation, and specific forms of WBH.

### Implications and recommendations for policy and practice

The findings of this study provide those with responsibility for safe working environments with the evidence to justify early intervention. This includes implications for policies at both the organisational and legislative level. National legislation is beneficial for holding organisations accountable in court, but leaders within organisations must develop and fairly apply anti-bullying policy. To take the first, unlike other European countries (e.g. Sweden and France), the UK has no legislation that directly addresses workplace bullying, in cases where it does not amount to unlawful harassment [18]. Instead, various other legal protections are relied upon (such as the Health and Safety at Work Act 1974, Employment Rights Act 1996, Protection from Harassment Act 1997 and the Equality Act, 2010), the implementation of which is patchy, piecemeal and ineffective.

In the absence of cohesive legislation, responsibility lies entirely with organisations. There are important implications of the current findings for workplaces - particularly for those in management and leadership positions and HR departments, whose direct responsibility is to protect employees. However, our findings indicate that WBH is perpetrated most by people with management responsibilities, and the potential for power dynamics to be at play must not be forgotten when tackling what happens in workplaces. Structural issues in the workplace can create pressure for managers which they then take out on those they manage [63], managers can be victims of WBH themselves [64], and organisational culture may perpetuate WBH [65]. Thus, there is a need for awareness raising and increased recognition of WBH at all levels, such that employees (either victims or witnesses) recognise what behaviours are bullying, employers and colleagues recognise when their own behaviour is bullying, and HR are sensitive to recognising the signs that it is happening. One promising avenue might be bystander intervention training [66]. As the direction of causality remains unclear, and poor mental health can predispose people to perceive bullying, there are also policy implications regarding the challenges of managing people in the workplace with poor mental health, including support for managers.

Developing policies and guidance for managers is challenging due to the need to define WBH. Providing a definition can help people recognise the behaviours ‘included’, but providing a list of examples or too tight a definition risks alienating those experiencing WBH in a different form. Furthermore, studies into the implementation of WBH policies and legislation suggest that most are either not adhered to, ineffective, or both [67], representing one of multiple barriers to reporting WBH [31]. Having a definition of WBH in UK legislation might help. Finally, HRs might not be the most approachable people for victims of WBH [68], further highlighting the need for alternative sources of support within organisations, such as unions and counselling services.

Mental health professionals need to be aware of the prevalence of WBH and potential for it to be a cause of a wide range of mental health issues, so they can provide effective treatment. The costs to individuals and society further highlights the need for organisations to intervene as soon as WBH is suspected. Over and above reactive measures, prevention efforts and early intervention are needed. Employers must take responsibility for developing and implementing preventative measures, responsive interventions as issues emerge, and rehabilitative approaches where harm has already been caused. Rather than prescribed ‘tick-box’ policies and responses, creative methods incorporating employee’s perspectives will likely be needed to make meaningful changes. This study has also shown that WBH may affect the financially disadvantaged most of all. This underscores the need for responses to WBH to pay attention to at-risk groups and the power dynamics involved.

The above discussion leaves several suggestions for future research. Despite consistent empirical evidence of the high prevalence and negative consequences of WBH, it is a challenging phenomenon to study, and existing studies are hampered by methodological limitations. This has also meant that cross-cultural comparisons are difficult to interpret. By analysing data from a population-based survey, the current study has made a valuable contribution to knowledge on WBH in the UK, and similarly representative studies are needed in other countries. Further, longitudinal, population-based studies that have good measures of WBH, include better measures of mental disorder, and adjust for potential covariates (both internal and external to the workplace) are needed to better understand the level of risk that WBH independently contributes to various mental health outcomes, and how long these impacts on mental health are felt. Such studies could also compare different occupations, particularly high-risk population groups, and consider underexplored areas such as sexual orientation and gender identity. Further analyses looking at variations in experience of WBH by precarious occupational or employment status is also needed, to address the finding that those in positions of ‘lower power’ are more likely to be exposed to bullying. Such work should also investigate the institutional structures (re)producing these inequalities. The intent of the perpetrator to cause harm is also often not considered in WBH research due to the challenges of empirically assessing intention [69] but this would be a valuable methodological development, alongside adopting a more victim-centred approach [16], as is the case in educational settings. Future research should take into account the context of changing workplaces, including emergent characteristics of remote working and increased surveillance.

## Conclusions

This study has provided urgently needed updated prevalence rates of WBH in UK workplaces. Our findings demonstrate that exposure to WBH is common, perhaps more so than previously reported, and that there are inequalities in exposure. Women are more likely to report WBH than men. Whilst the impact of bullying on women’s mental health is no different than for men, there are gender differences in the types of bullying behaviours experienced. Other groups disproportionately affected are ethnic minorities and the financially disadvantaged. Perhaps relatedly, WBH is also most often perpetrated by people in power (i.e. managers), and this power dynamic should not be forgotten when addressing issues in the workplace. Our findings further show that WBH is strongly associated with mental health, and the harms of exposure can pervade every aspect of a person– cognitive, behavioural and relational. Given pandemic-related and other major changes in workplace practices in recent years, the need to understand and develop more effective responses to workplace bullying has never been greater, and it is hoped that this study will inspire future research on the topic.

## Electronic supplementary material

Below is the link to the electronic supplementary material.

Supplementary Material 1

## Data Availability

The datasets analysed during the current study are available from the UK Data Service repository [https://beta.ukdataservice.ac.uk/datacatalogue/series/series?id=200004410.5255/UKDA-Series-2000044]. Restrictions apply to the availability of these data, which were used under license for the current study, and so are not publicly available. Data are however available from the UK Data Service with permission of [Data Access Request Service at NHS England https://digital.nhs.uk/services/data-access-request-service-dars].

## Abbreviations

WBH: workplace bullying and harassment
HR: human resources
PTSD: post-traumatic stress disorder
GHQ-12: General Health Questionnaire
APMS: Adult Psychiatric Morbidity Survey
CAPI: Computer-assisted personal interviewing
CASI: Computer-assisted self-completion interview
CMD: Common mental disorder
CIS-R: Clinical Interview Schedule– Revised
ICD-10: International Classification of Diseases
DSM-IV: Diagnostic and Statistical Manual of Mental Disorders, 4th Edition
WEMWBS: Warwick Edinburgh Mental Wellbeing Scale
