# Correction: Prevalence and nature of workplace bullying and harassment and associations with mental health conditions in England: a cross-sectional probability sample survey

**DOI:** 10.1186/s12889-026-28010-y

**Published:** 2026-06-16

**Authors:** Annie Bunce, Ladan Hashemi, Charlotte Clark, Stephen Stansfeld, Carrie-Anne Myers, Sally McManus

**Affiliations:** 1 Violence and Society Centre, City, University of London, Rhind Building, Northampton Square, London, EC1V 0HB UK; 2 Population Health Research Institute, St George’s, University of London, Cranmer Terrace, London, UK; 3 Centre for Psychiatry and Mental Health, Barts & the London School of Medicine, Queen Mary University of London, London, UK; 4 Department of Sociology and Criminology, School of Policy and Global Affairs, City, University of London, London, EC1V 0HB UK; 5 National Centre for Social Research, 35 Northampton Square, London, EC1V 0AX UK

**Correction: BMC Public Health 24**,** 1147 (2024)**

**https://doi.org/10.1186/s12889-024-18614-7**

Following publication of the original article [1], the authors reported an error in Table 1. The heading ‘WBH experienced in past year’ needs to be swapped with the heading ‘No WBH in past year’. The (*n* = 3394) relates to ‘No WBH in past year’, and the (*n* = 444) relates to ‘WBH experienced in past year’.

The original article [1] has been updated.
