# Supplementary material for: Prevalence and nature of workplace bullying and harassment and associations with mental health conditions in England: a cross-sectional probability sample survey
Source: BMC Public Health. 2024 Apr 24;24:1147. doi: 10.1186/s12889-024-18614-7 (PMC11044501; doi:10.1186/s12889-024-18614-7)
Supplement: Supplementary file 1 — Supplementary Material 1 [file 12889_2024_18614_MOESM1_ESM.docx]

**Supplementary Table 1. Definition of workplace bullying and harassment, poor mental health and mental wellbeing, the 2014 England Adult Psychiatric Morbidity Survey**

| **Variable** |  |
| --- | --- |
| **Bullying** | Have you personally experienced bullying or harassment at work in the last twelve months? Yes/No |
| **If bullied** | Who was the person, or people, responsible?  1 Your line manager or another manager  2 A colleague  3 A member of Human Resources  4 A student  5 Client or a customer  6 Member (s) of the public  7 Other (give details) |
| **If bullied** | What form does or did the bullying take?  1 Threatening behaviour  2 Shouting or verbal abuse  3 Physical abuse  4 Humiliation  5 Excessive Criticism  6 Constantly changing instructions  7 Excessive workloads  8 Setting unrealistic targets  9 Refusing reasonable requests (i.e. for leave or training)  10 Sexual harassment  11 Cyber bullying  12 Other (please specify) |
| **Poor mental health** | Somatic symptoms: respondents were asked if they have suffered from any discomfort such as any sort of ache or pain in the past month |
|  | Fatigue: they noticed that they have been getting tired in the past month |
|  | Concentration problem: if they have had any problems in concentrating on what they were doing in the past month |
|  | Sleep problems: if they have been having problems with trying to get to sleep or with getting back to sleep if they woke up or were woken up in the past month |
|  | Irritability: if they have felt irritable or short tempered with those around them in the past month |
|  | Worried about physical health: If they have been at all worried about their physical health, in the past month |
|  | Feeling depressed: if they have had a spell of feeling sad, miserable or depressed in the past month |
|  | Being worried: if they found themselves worrying about things more than they needed to, in the past month |
|  | Feeling anxious: If they have been feeling anxious or nervous in the past month |
|  | Compulsion: if they found that they kept on doing things over and over again when they knew they had already done them. For example, making their bed or washing their hands over and over again in the past month |
|  | Obsessive thoughts: if they had any thoughts or ideas over and over again that they found unpleasant and would prefer not to think about, that still kept on coming into their mind in the past month. For example, constantly thinking about death. |
|  | Any common mental disorder (CMD): any of six depressive and anxiety disorders: generalised anxiety disorder, phobic disorder, panic disorder, obsessive and compulsive disorder, depression, and CMD not otherwise specified. (past week): neurotic (CISR). |
| **Mental wellbeing** | Feeling optimistic: I've been feeling optimistic about the future |
|  | Feeling useful: I've been feeling useful |
|  | Feeling relaxed: I've been feeling relaxed |
|  | Feeling interested in other people: I've been feeling interested in other people. |
|  | Had energy to spare: I've had energy to spare |
|  | Dealing with problems well: I've been dealing with problems well |
|  | Thinking clearly: I've been thinking clearly |
|  | Feeling good about themselves: I've been feeling good about myself |
|  | Feeling close to other people: I've been feeling close to other people |
|  | Feeling confident: I've been feeling confident |
|  | Able to make up own mind about things: I've been able to make up my own mind about things. |
|  | Feeling loved: I've been feeling loved. |
|  | Interested in new things: I've been interested in new things. |
|  | Feeling cheerful: I've been feeling cheerful. |
